# Validity of a measure to assess healthy eating and physical activity policies and practices in Australian childcare services

**DOI:** 10.1186/1471-2458-14-572

**Published:** 2014-06-09

**Authors:** Pennie Dodds, Rebecca Wyse, Jannah Jones, Luke Wolfenden, Christophe Lecathelinais, Amanda Williams, Sze Lin Yoong, Meghan Finch, Nicole Nathan, Karen Gillham, John Wiggers

**Affiliations:** 1 Faculty of Health, School of Medicine and Public Health, The University of Newcastle, Newcastle, NSW 2300, Australia; 2 Hunter New England Population Health, Hunter New England Local Health District, Locked Bag No. 10, Newcastle, Wallsend NSW 2287, Australia; 3 Priority Research Centre for Health Behaviour, The University of Newcastle, Newcastle, NSW 2308, Australia; 4 Hunter Medical Research Institute, Newcastle, NSW 2300, Australia

**Keywords:** Healthy eating, Physical activity, Obesity prevention, Childcare, Nursery, Preschool, Measurement tool

## Abstract

**Background:**

Childcare services represent a valuable obesity prevention opportunity, providing access to a large portion of children at a vital point in their development. Few rigorously validated measures exist to measure healthy eating and physical activity policies and practices in this setting, and no such measures exist that are specific to the childcare setting in Australia.

**Methods:**

This was a cross sectional study, comparing two measures (pen and paper survey and observation) of healthy eating and physical activity policies and practices in childcare services. Research assistants attended consenting childcare services (n = 42) across the Hunter region of New South Wales, Australia and observed practices for one day. Nominated Supervisors and Room Leaders of the service also completed a pen and paper survey during the day of observation. Kappa statistics and proportion agreement were calculated for a total of 43 items relating to healthy eating and physical activity policies and practices.

**Results:**

Agreement ranged from 38%-100%. Fifty one percent of items showed agreement of greater than or equal to 80%. Items assessing the frequency with which staff joined in active play with children reported the lowest percent agreement, while items assessing availability of beverages such as juice, milk and cordial, as well as the provision of foods such as popcorn, pretzels and sweet biscuits, reported the highest percent agreement. Kappa scores ranged from −0.06 (poor agreement) to 1 (perfect agreement). Of the 43 items assessed, 27 were found to have moderate or greater agreement.

**Conclusions:**

The study found that Nominated Supervisors and Room Leaders were able to accurately report on a number of healthy eating and physical activity policies and practices. Items assessing healthy eating practices tended to have higher kappa scores than those assessing physical activity related policies or practices. The tool represents a useful instrument for public health researchers and policy makers working in this setting.

## Background

More than 43 million preschool aged children worldwide are classified as overweight or obese [1]. The prevalence of child overweight and obesity has doubled over recent decades with an estimated 20-25% of Australian preschool aged children currently overweight or obese [2,3]. Targeting obesity prevention during early childhood has been recommended as an important public health strategy to reduce the burden of future chronic disease [4]. As such, internationally, and in Australia, governments have invested in initiatives to promote healthy eating and physical activity among children [5-7].

Centre based childcare services provide a valuable obesity prevention opportunity, given this setting provides education and care to a significant proportion of children aged 3–5 years [8,9] at a vital point in their development [10]. In countries such as Australia, the United States and the United Kingdom, over 50% of children aged under five years attend some form of centre based childcare service, often for prolonged periods of time [11-13]. Childcare services also have existing infrastructure to support obesity prevention initiatives and staff are amenable to programs which seek to improve children’s diet and encourage child physical activity [14,15]. Furthermore, Australian childcare services are required to adhere to licensing and accreditation requirements, a number of which promote the health and physical development of children [7].

Given evidence of the positive impact that the childcare service environment can have on diet quality [16-18], physical activity [19], fundamental movement skill (FMS) proficiency [18] and obesity prevention [17], Australian best practice guidelines for the childcare service setting recommend the implementation of a number of practices, including; written nutrition and physical activity policies, provision of water and age appropriate milk, increasing opportunities for structured physical activity, encouraging role modelling surrounding healthy eating and physical activity, limiting sedentary screen time and partnering with families to encourage healthy eating and physical activity [20]. To determine the extent to which such guidelines are adopted, valid measures of the implementation of obesity prevention policies and practices are required. While direct observation is generally considered the ‘gold standard’ for environmental assessment [21-23] at a population level the collection of such data is expensive and impractical, impeding policy development and research in this setting.

To our knowledge, two survey instruments have been validated which may be used for population based assessment of the obesity prevention characteristics of childcare services. Both instruments were developed for use in the United States. The first is the Nutrition and Physical Activity Self-Assessment for Child Care assessment tool (NAP SAAC) [24,25], a self-administered survey that allows services to evaluate their own nutrition and physical activity environment. The NAP SAAC tool was validated by direct observation and many items in the tool were demonstrated to provide a measure of the nutrition and physical activity environment of childcare services. Kappa statistics ranged from −0.01 to 0.79, with the highest validity shown for questions relating to nutrition and physical activity policy. Similarly, the Child Care Nutrition and Physical Activity Assessment Survey tool [26] was assessed by direct observation and interview, and showed reasonably high agreement for questions relating to policy and nutrition [26].

While both instruments represent useful tools for both practitioners and researchers to assess the obesity prevention characteristics of childcare services in the United States, regulatory, operational, environmental and cultural differences between countries limits the utility of these tools for use in other countries. For example, national recommendations regarding diet and physical activity for children aged under five years differ between the United States and Australia [27,28], as do key operational characteristics of services such as staffing ratios and child health accreditation requirements [29,30].

While numerous studies in Australia have assessed the obesity prevention policies and practices of childcare services via surveys of childcare service staff [31-34], to our knowledge none of these tools have been validated. As such, the aim of this study was to develop and assess the validity of a survey tool to assess healthy eating and physical activity policies and practices in Australian childcare services.

## Methods

Approval to conduct the study was obtained from Hunter New England Local Health District (12/08/15/5.01) and The University of Newcastle Human Research Ethics Committees (H-2012-0321).

### Design & setting

This cross sectional study compared two measures (pen and paper survey and observation) of healthy eating and physical activity policies and practices of childcare services. The study took place in the Hunter region of New South Wales, Australia. This region encompasses non-metropolitan ‘major cities’ and ‘inner regional’ areas as described by the Australian Statistical Geography Standard [35]. Approximately 3% of residents are of Aboriginal or Torres Strait Islander origin and 4% speak languages other than English [36,37].

### Sample

For the purpose of the study centre based childcare services were defined as preschools and long day care services. In New South Wales, preschools provide centre based care for six to eight hours per day and enrol children aged between three to six years. Long day care services provide centre based care for eight or more hours per day and usually enrol children aged from six weeks old up to six years. Both types of services provide specific programs for children aged three to five years that provide educational and developmental activities to assist children in their preparation for school [8]. Across Australia the role and function of preschools and long day care services are similar and licensing and accreditation requirements regarding healthy eating and physical activity policies and practices are identical [7]. Furthermore implementation of physical activity practices in these settings is similar [38].

A database of all preschool and long day care services (hereafter referred to as services) located within the study region was generated from a list provided by the State Office of Childcare from the Department of Community services. Eligible services were those located within the Hunter region who have children aged 3–5 years in their care, and whose families provide all or some of the food for children. A random sample of 80 eligible services (approximately 22% of all services in the study region), were invited to participate in the study.

### Recruitment

The Nominated Supervisor (service manager) of each service was sent an information letter and consent form, inviting them to participate in the study. Services were asked to post or email back signed consent forms. Two weeks following receipt of the invitation, non-responders were telephoned by a research assistant to assess interest in participation. Following consent, a research assistant telephoned the service to schedule a day for the site visit and surveys to be completed. The study was conducted from November 2012 to March 2013, with all surveys and site visits completed between January and March 2013.

### Data collection procedures

#### Written surveys

Two pen and paper surveys were developed to evaluate healthy eating and physical activity policies and practices of services. The first survey was designed to be completed by the Nominated Supervisor (or service manager), the person responsible for the management of the service. This survey included items assessing whole-of-service policies and practices. The second survey was designed to be completed by the Room Leader of a 3–5 year old room, typically the head teacher of the classroom. The Room Leader written survey included items to assess specific healthy eating and physical activity policies or practices of their room.

The written survey items were constructed following a review of the literature and were based on a number of existing tools including the United States Nutrition and Physical Activity Self- Assessment for Child Care [24,25] and the Child Care Nutrition and Physical Activity Assessment Survey [26], together with surveys previously developed and implemented by the research team [32,34,38] and regulations surrounding childcare service policies and practices associated with healthy eating, physical activity or obesity prevention [7]. Survey items were informally piloted with a convenience sample of Nominated Supervisors and Room Leaders together with health promotion practitioners to assess comprehension and understanding. The survey items were then amended based on the feedback received.

The Nominated Supervisor and Room Leader of each service were asked to complete the relevant survey during a site visit.

#### Observational site visit

An observation checklist was developed for the purposes of a one-day site visit conducted at every service, and was constructed based on items included in the written surveys. The observation checklist consisted of questions surrounding: observational records, service characteristics, meals and beverages, role modelling, structured physical activity, fundamental movement skills, physical activity during free play and small screen recreation.

All research assistants attended a two hour training session conducted by the research team prior to the commencement of site visits. To ensure standardisation, research assistants were required to complete a quasi-observation assessment from information provided in hypothetical case studies. Responses were discussed with research assistants after completion.

The observational site visit was conducted for every service by a trained research assistant from January to March of 2013 and took place over one full day of service operating hours (minimum of 9 am-3 pm). During the site visit, research assistants observed service practices and completed the observation checklist. The observation checklist was completed as events occurred. Research assistants passively observed service practices from a location which did not interfere with normal practice. In addition to the observation checklist, research assistants that completed the observational site visits also arranged a short 10 minute meeting with Nominated Supervisors to collect information and copies of policy documents relating to healthy eating and physical activity.

### Measures

Items within the written surveys were as follows:

#### Healthy eating practices

(a) Child beverages - Room Leaders were asked to report (yes /no) if any of the following drinks were provided at the service: cordial; flavoured milk; fruit juice; water; plain milk; or soft drink. Nominated Supervisors were asked to report on the type of milk served to children (regular/reduced fat).

(b) Child food – Nominated Supervisors were asked to report (yes/no) if any of the following foods were provided at the service: confectionary/chocolate/ice-cream; fruit/vegetables; pretzels/plain popcorn/oven baked chips; sweet biscuits.

(c) Lunchbox monitoring - Room Leaders were asked to report (yes/no) if they monitor lunchboxes; provide feedback to families about lunchbox contents and whether parents provided any food not compliant with service nutritional guidelines on the day of the visit.

(d) Role modelling - Room Leaders were asked to report (yes/no) if staff members consume any of the following in front of the children: fruit; vegetables; sweets, salty or sugary snacks or drinks and if so, how many staff consume each of these food items in front of the children (all staff/fewer staff). Room Leaders were also asked to report if educators provide positive comments about healthy foods to children (yes/no).

#### Physical activity practices

(a) Free play - Room Leaders were asked to report how much time (less than 4.5 hours/4.5 hours or more) children spend in free play and how much time is spent in physically active free play (less than 2.5 hours/2.5 hours or more). They were also asked to report the average proportion (less than or equal to 75%/more than 75%) of children who participate in activity during free play. Active free play was defined as where children were moving their body from one location to another, or if standing, moving at least their limbs and trunk.

(b) Role modelling - Room Leaders were asked to report (yes/no) if educators participate alongside children in active free play and provide verbal prompts to encourage the children’s activity, the number of educators that do this (all staff /fewer staff) and how often educators join in with the children (never or sometimes/often or always).

(c) Structured educator led physical activity- Room Leaders were asked to report (yes/no) if structured educator led physical activity was included on the day of the visit, how much time (<30 min/30 min or more) children usually have in structured educator led physical activity and the average proportion (less than or equal to 90%/more than 90%) of children usually active during structured educator led physical activity. Additionally, they were asked to report if structured educator led physical activity to develop fundamental movement skills was included on the day of the visit (yes/no) and the average proportion (less than or equal to 96%/more than 96%) of children usually active during fundamental movement skills sessions.

(d) Small screen recreation- Room Leaders and Nominated Supervisors were asked to report (yes/no) whether DVDs and videos or computers and tablets are available for children to use, and if so for what purpose these were used (active vs not active).

#### Policies

Nominated Supervisors were asked to provide copies of written nutrition, physical activity, and/or small screen recreation policies as well as written nutritional guidelines for families to observers at the conclusion of the site visit (yes/no).

### Analysis

#### Descriptive statistics

Statistical analyses were conducted in the statistical software program SAS version 9.3 (SAS Institute Inc., Cary, NC, USA). Descriptive statistics were used to describe the characteristics of participating childcare services. Socio-Economic Index for Australia (SEIFA index) was calculated based on service postcodes. The SEIFA index was categorised into tertiles and used to classify services as located in a low, medium or high socio-economic area [39].

#### Validity

Items included in the observation checklist were developed to correspond with items from the written surveys. In both tools, continuous items were dichotomised based on the median value, and categorical variables were collapsed into two categories prior to analysis. Two methods of examining validity of written surveys were reported; percentage agreement and Cohen’s kappa [40]. Where written surveys were not returned by services, data was recorded as missing. Percentage agreement greater than 80% was considered evidence for strong agreement [41]. Kappa is reported in addition to percentage agreement as the statistics adjusts for agreement due to chance [42]. Consistent with previous research [43], where positive agreement accounted for over 75% or under 25% of total agreement, prevalence adjusted and bias adjusted kappa was reported (PABAK) [44,45]. Based on benchmarks suggested by Landis and Koch [46], agreement for kappa measures were classified in the following categories: poor = <0; slight = 0.00-0.20; fair = 0.21-0.40; moderate = 0.41-0.60; substantial = 0.61-0.80; almost perfect = 0.81-1.00.

## Results

### Sample

A total of 80 services in the Hunter region were approached to participate in the study and 42 services consented to participate (52.5% consent rate). All services returned the Room Leader survey, and 40 services (95%) returned the Nominated Supervisor survey. A one day observational site visit was conducted for all services.

### Descriptive statistics

Services were open for an average of 9.1 hours, had on average 85 total enrolments and 22 3–5 year old children present on the day of the observation (Table 1). The majority of services were long day care services (61.9%). Services located in lower SEIFA index categories were less likely to consent to participate in this study (*p* = 0.02). There were no other significant differences in the operational characteristics of services that did and did not consent to participate.

**Table 1:** Descriptive statistics of study sample

***Measure***	***Category***	***Count (Percentage)***
		***N = 42***
Socio-economic disadvantage (SEIFA)	Low	9 (21.4%)
	Medium	29 (69.1%)
	High	4 (9.5%)
Service type^a^	Preschool	17 (40.5%)
	Long day care service	26 (61.9%)
Number of allocated places^b^	1-39	26 (61.9%)
	40-190	16 (38.1%)
Number of enrolments^b^	1-80	24 (57.1%)
	81-200	18 (42.9%)
Number of educators^b^	1-8	24 (58.5%)
	9-23	17 (41.5%)
Number of days open^b^	5 Days	37 (88.1%)
	4 days or less	5 (11.9%)
Hours open^b^	<10 hours	21 (50.0%)
	> = 10 hours	21 (50.0%)

^a^Percentages do not add up to 100% as some services reported being a combination of different types.

^b^Median response is used as category cut-off point.

### Validity

A total of 43 items were assessed by comparing responses for pen and paper surveys to observation records.

#### Percentage agreement

Agreement ranged from 38% to 100%. A total of 22 (51%) written survey items showed agreement greater than or equal to 80%, and 35 (81%) with agreement greater than or equal to 60%. Items assessing the frequency which staff joined in active play with children reported the lowest percent agreement while items assessing availability of beverages such as juice, milk and cordial, as well as the provision of foods such as popcorn pretzels and sweet biscuits reported the highest percent agreement.

#### Kappa

Kappa scores ranged from −0.06 (poor agreement) to 1 (perfect agreement). A total of two items (6%) were considered to have poor agreement; seven (19%) to have slight agreement; seven (19%) to have fair agreement; six (17%) to have moderate agreement; nine (25%) to have substantial agreement and twelve (33%) to have almost perfect agreement.

Items assessing healthy eating practices tended to have higher kappa scores than those assessing physical activity related policies or practices.

PABAK was calculated for a total of 36 of the 43 items assessed, as each had a positive agreement of greater than or equal to 75%, or less than or equal to 25%. For all remaining survey items (where positive agreement 26%-74%), kappa was calculated (Table 2).

**Table 2:** Validity of written survey items

**Category**	**Label**	**Written survey source**	**Response levels**	**Sample**	**Percentage agreement**	**Kappa**	**95% lower CI**	**95% upper CI**	**Validity assessment**
**Physical activity**									
Free play	How much of the daily operating time, do 3 to 5 year old children have for child-initiated free play^b^	Room Leader	2 (<4.5 hrs/> = 4.5 hrs)	40	63%	0.25	−0.05	0.55	Fair
	For how much of the child-initiated, free play time can children be physically active^b^	Room Leader	2 (<2.5 hrs/> = 2.5 hrs)	40	55%	0.1a	−0.21	0.41	Slight
	Average proportion of children active during free play time	Room Leader	2 (<=75%/>75%)	38	66%	0.32^a^	0.01	0.62	Fair
Small screen recreation	Were computers or tablets used by children aged 3 to 5	Room Leader	2 (yes/no)	35	77%	0.54^a^	0.26	0.83	Moderate
	Were television (TV), videos and DVDs, including educational programs and videos, viewed by children aged 3 to 5	Room Leader	2 (yes/no)	36	94%	0.89^a^	0.74	1.04	Almost Perfect
	Purpose for watching TV/videos	Nominated Supervisor	2 (active vs not active)	12	58%	0.17^a^	−0.42	0.75	Slight
Structured educator led physical activity	Did educators in the 3–5 room provide structured, educator-led physical activity	Room Leader	2 (yes/no)	35	94%	0.89a	0.73	1.04	Almost Perfect
	How much time spent in structured educator led physical activity^b^	Room Leader	2 (<30 min/> = 30 min)	37	54%	0.08a	−0.24	0.41	Slight
	Average proportion of children active during structured, educator led physical activity^b^	Room Leader	2 (<90%/> = 90%)	23	52%	0.04^a^	−0.37	0.46	Slight
Structured educator led Fundamental Movement Skills (FMS)	Did educators in this room lead structured activity to develop FMS	Room Leader	2 (yes/no)	34	53%	0.06a	−0.28	0.4	Slight
	Average proportion of children active during structured, educator-led physical activity to develop FMS^b^	Room Leader	2 (<96%/> = 96%)	10	60%	0.2	−0.36	0.76	Slight
**Healthy eating**									
Child beverages	Cordial available	Room Leader	2 (yes/no)	36	100%	1^a^	1	1	Almost Perfect
	Flavoured milk available	Room Leader	2 (yes/no)	36	100%	1^a^	1	1	Almost Perfect
	Fruit juice available	Room Leader	2 (yes/no)	36	100%	1^a^	1	1	Almost Perfect
	Only water and/or plain milk available	Room Leader	2 (yes/no)	33	100%	1^a^	1	1	Almost Perfect
	Plain milk available	Room Leader	2 (yes/no)	36	94%	0.88	0.73	1	Almost Perfect
	Type of milk served to children	Nominated Supervisor	2 (regular vs reduced fat)	14	79%	0.57a	0.13	1.02	Moderate
	Soft drink available	Room Leader	2 (yes/no)	36	100%	1^a^	1	1	Almost Perfect
	Water available	Room Leader	2 (yes/no)	36	89%	0.78^a^	0.57	0.99	Substantial
Child food	Only Healthy Foods offered	Nominated Supervisor	2 (yes/no)	11	73%	0.45	-.10	1.01	Moderate
	Confectionary, chocolate, ice cream	Nominated Supervisor	2 (yes/no)	14	86%	0.71	0.33	1.09	Substantial
	Fruit or vegetable pieces, salad or platters	Nominated Supervisor	2 (yes/no)	14	93%	0.86	0.58	1.14	Almost Perfect
	Pretzels, plain popcorn or oven-baked chips (not oiled)	Nominated Supervisor	2 (yes/no)	14	100%	1	1	1	Almost Perfect
	Sweet biscuits with chocolate or cream filling	Nominated Supervisor	2 (yes/no)	14	100%	1	1	1	Almost Perfect
	No foods	Nominated Supervisor	2 (yes/no)	14	86%	0.71	0.33	1.09	Substantial
Lunchbox monitoring	Do educators monitor lunchboxes?	Room Leader	2 (yes/no)	32	84%	0.69a	0.43	0.94	Substantial
	Do educators monitor lunchboxes and provide feedback to families?	Room Leader	2 (yes/no)	25	68%	0.34	−0.03	0.71	Fair
	Did parents provide any foods that weren’t compliant with guidelines	Room Leader	2 (yes/no)	21	86%	0.71^a^	0.41	1.02	Substantial
**Role modelling**									
Food & drink	Did staff members consume fruit in front of children	Room Leader	2 (yes/no)	41	88%	0.76^a^	0.55	0.96	Substantial
	How many staff consumed fruit in front of children	Room Leader	2 (all vs fewer staff)	40	68%	0.35a	0.06	0.64	Fair
	Did staff members consume vegetables in front of children	Room Leader	2 (yes/no)	41	49%	−0.02^a^	−0.33	0.29	Poor
	How many staff consumed vegetables in front of children	Room Leader	2 (all vs some staff)	41	88%	0.76^a^	0.55	0.96	Substantial
	Did educators make positive comments about healthy foods	Room Leader	2 (yes/no)	35	86%	0.71^a^	0.48	0.95	Substantial
	Did staff consume sweets, salty or sugary snacks or drinks in front of children	Room Leader	2 (yes/no)	35	94%	0.89^a^	0.73	1.04	Almost Perfect
Active free play	Did educators from this room participate alongside children in active play	Room Leader	2 (yes/no)	36	69%	0.39^a^	0.08	0.69	Fair
	How many educators participated alongside children in active play	Room Leader	2 (all vs fewer staff)	41	61%	0.22a	−0.08	0.52	Fair
	How often did educators join in with children in active play	Room Leader	2 (Never or Sometimes vs often or always)	40	38%	−0.06	−0.28	0.16	Poor
	Did educators provide verbal prompts to encourage child activity	Room Leader	2 (yes/no)	35	80%	0.6^a^	0.33	0.87	Moderate
	How many educators provided prompts to encourage child activity	Room Leader	2 (all vs fewer staff)	34	62%	0.24^a^	−0.1	0.57	Fair
**Policy**									
	Written small screen recreation policy	Nominated Supervisor	2 (yes/no)	35	89%	0.77	0.56	0.98	Substantial
	Written physical activity policy	Nominated Supervisor	2 (yes/no)	38	79%	0.59	0.36	0.82	Moderate
	Written nutrition policy	Nominated Supervisor	2 (yes/no)	40	75%	0.5^a^	0.23	0.77	Moderate
	Written nutritional guidelines for families	Nominated Supervisor	2 (yes/no)	35	54%	0.09a	−0.25	0.42	Slight

^a^PABAK is reported where positive agreement > =75% or < =25%.

^b^Item required continuous response.

## Discussion

This study sought to develop and validate a survey tool assessing the healthy eating and physical activity policies and practices of Australian childcare services. The findings of the study suggest that Nominated Supervisors and Room Leaders were able to accurately report a number of policies and practices using the tool. Such findings suggest that the tool may provide a valid means of assessing some healthy eating and physical activity practices of services and represent a useful instrument for public health researchers and policy makers working in this setting.

Of the 43 items assessed, 27 were found to have moderate or greater agreement. Moderate or greater agreement was found for healthy eating practices with 16 of 17 items. Among the most accurately reported healthy eating practices were the availability of food and beverages for children at the service, where percent agreement ranged from 73-100% and kappa from 0.45-1. Such measures of agreement are higher than previous validation studies comparing reports of childcare service staff with direct observation or document review [23,24]. These studies report percent agreement ranges between 33%-94% and kappa between 0.23-0.40 for the availability of beverages including water, fruit juice and sweetened drinks. The differences in validity between studies may represent differences in operational characteristics of the study samples.

In contrast to child food and beverage provision, only three of 11 items assessing physical activity practices were found to have moderate or greater agreement. This may be due to the type of response option required; six of the eight items that showed less than moderate agreement required either a time or percentage estimate (as opposed to a categorical response option such as yes/no, characteristic of the food and beverage items above). Each of these items resulted in only fair-slight agreement, with a mean kappa rating of 0.17 (58%). In comparison, the mean kappa rating for survey items relating to physical activity that required categorical response options was 0.51 (75%). Further refinement of this tool could include the removal of such response options in favour of more discrete, dichotomous outcomes (e.g. yes/no). In addition, the validity of Room Leader reports of behaviour of their staff to encourage child physical activity, such as participation in activity alongside children and the provision of verbal prompts was variable. For the five items which assessed such practices, percent agreement ranged from 38-80% and kappa from −0.06-0.60. A single item in a previous study assessing staff participation with children in active play yielded a percent agreement of 70% and a Kappa of 0.59 [23]. Surveying individual staff regarding their own behaviours, may provide more valid data regarding staff engagement in these physical activity promoting practices.

A number of study limitations are worth noting. The study did not assess all characteristics of childcare services associated with the prevention of childhood obesity, such as the existence of fixed and portable play equipment and space available for physical activity. Previous research however, has suggested that such environmental factors are reported with high agreement [23]. Second, observation data were collected during a single day. Observation over a longer period may provide a more robust measure of practice implementation. Third, the sample of childcare services may not be representative of other service types in Australia (for example, those that supply meals), or internationally. Fourth, the measure of socio-economic disadvantage was calculated based on service postcodes. Given families may not reside in the same postcode as the service, this may not accurately reflect the true socio-economic characteristics of the service. Fifth, the completion of the written survey at the same time as the site visit may have influenced service practice and/or the practices reported by participants. Future validation studies should investigate the potential of covert observations or recordings which are likely to provide more robust estimates of validity.

## Conclusions

Notwithstanding these limitations the current study is the first assessment of childcare service implementation of healthy eating and physical activity policies and practices outside of the United States and includes the assessment of practices not previously validated, such as assessment of practices relating to child lunchboxes. Given the cost of direct observation, generally considered as the gold standard for such environmental assessments of service delivery settings, the study provides researchers with a valid and relatively inexpensive means to conduct research to describe childcare service environments and assess the impact of obesity prevention initiatives. Similarly, the tool may prove useful for health practitioners and policy makers interested in monitoring childcare implementation of healthy eating and physical activity policies and practices and in identifying services that require support to implement specific policies and practices.

## Competing interests

Authors have no competing interests to declare.

## Authors’ contributions

PD, LW and RW contributed to the conception and design of the project. PD and RW were responsible for the acquisition of data. PD and CL were responsible for the analyses of data. PD coordinated manuscript preparation and all authors contributed to the interpretation of data, manuscript preparation and critical revisions. All authors read and approved the final manuscript.

## Pre-publication history

The pre-publication history for this paper can be accessed here:

http://www.biomedcentral.com/1471-2458/14/572/prepub
